# Proanthocyanidin Accumulation and Biosynthesis Are Modulated by the Irrigation Regime in Tempranillo Seeds

**DOI:** 10.3390/ijms150711862

**Published:** 2014-07-04

**Authors:** Tania Genebra, Raquen Raissa Santos, Rita Francisco, Marta Pinto-Marijuan, Ricard Brossa, Ana Teresa Serra, Catarina M. M. Duarte, Maria Manuela Chaves, Olfa Zarrouk

**Affiliations:** 1 Instituto de Tecnológia Quimica e Biologica, University Nova of Lisbon, Oeiras 2780-901, Portugal; E-Mails: taniag@itqb.unl.pt (T.G.); raissa@itqb.unl.pt (R.R.S.); ritaf@itqb.unl.pt (R.F.); tserra@itqb.unl.pt (A.T.S.); cduarte@ itqb.unl.pt. (C.M.M.D.); mchaves@itqb.unl.pt (M.M.C.); 2 Departament of Biologia Vegetal , Facultat de Biologia, Barcelona University, Barcelona 6450-8028, Spain; E-Mails: marta.pinto.marijuan@gmail.com (M.P.-M.); brossa55@hotmail.com (R.B.); 3 IBET, Instituto de Biologia Experimental e Tecnológica, Apartado 12, Oeiras 2781-901, Portugal

**Keywords:** antioxidant capacity, gene expression, regulated deficit irrigation, tannin, *Vitis vinifera*, water stress

## Abstract

The main effects of three different irrigation regimes, *i.e*., sustained deficit irrigation (SDI), regulated deficit irrigation (RDI) and non-irrigated (NI), on seed traits namely proanthocyanidins (PAs) were evaluated in the wine grape cultivar Aragonez (syn. Tempranillo) grown in Alentejo (Portugal) over two growing seasons. Results showed that while the number of seeds per berry was not affected by water availability, seed fresh weight differed among treatments, the NI treatment exhibiting the lowest values. The biosynthetic pathway of flavanols appeared to be modified by the irrigation treatment, and several genes responsible for PA synthesis were up-regulated in the most stressed seeds (RDI and NI). However, this effect had no impact on PA content, suggesting the influence of other factors such as oxidation and/or degradation of PAs at late stages of maturation in grape seeds. The seeds’ non-enzymatic antioxidant capacities (oxygen radical absorbance capacity (ORAC) and hydroxyl radical adverting capacity (HORAC)) were modulated by water deficit and correlated well with PA content. The impact of irrigation strategy on PA biosynthesis, content, and anti-radical activity during seed ripening is discussed in the context of increasing interest in the role of PAs in the color and taste of wine, and the potential health benefits relating to their antioxidant capacity.

## 1. Introduction

Proanthocyanidins (PAs), also known as condensed tannins, are secondary metabolites synthesized via the flavonoid biosynthetic pathway. They are essentially oligomers and polymers of flavan-3-ol units and are widespread throughout the plant kingdom. PAs accumulate in many different organs and tissues [1] and present diverse biological and biochemical activities [2]. In wine grape, PAs are one of the main phenolic compounds conferring organoleptic properties, chiefly bitterness and astringency, hence significantly influencing taste [3]. They are mainly located in seeds, the source of approximately 50% of the flavan-3-ols in red wine [4,5]. In addition, studies on the effects of eliminating or adding seeds during winemaking showed their crucial role in stabilizing wine colour [6].

Depending on the grape variety, the monomeric flavan-3-ols are present in variable amounts also in relation to different stages of fruit ripening [7,8,9,10]. Generally, the majority of flavan-3-ol monomers accumulate prior to veraison and decrease thereafter, affecting tannin structure [7,8]. Seed tannins are oligomers and polymers composed of monomeric flavan-3-ols; (+)-catechin (C), (−)-epicatechin (EC), and (−)-epicatechin gallate (ECG). Their quantity has been found to change during the ripening process in some studies, but there are also reports to the contrary [7,9,11]. The oxidation of PAs that usually takes place after veraison causes the seed coat to change from bright green to dark brown [12].

In spite of the importance of PAs in red wine grapes, their biosynthetic pathway was only revealed during the last decade and is still only partially understood [13,14,15]. Flavanol monomers are formed by two biosynthetic routes, from either leucoanthocyanidins or anthocyanidins. Production of catechins from leucoanthocyanidins is catalysed by leucoanthocyanidin reductase (LAR), while anthocyanidin reductase (ANR) catalyses production of epicatechins from anthocyanidins [13,16]; however, it is still unclear whether the polymerization of PAs can occur spontaneously [1,2,17]. In grapevine, one isoform of ANR [15] and two LAR isoforms [14] were characterized in different grape berry tissues. As in other secondary metabolism pathways, the biosynthetic pathway of PAs is under complex control by multiple regulatory genes at the transcriptional level, namely *MYB* genes. In grape berry, *VvMYBPA1* was reported to specifically control *LAR* and *ANR* genes [18].

Grape seed PAs have beneficial effects on human health due to anticancer activities, antioxidant, cardio-protective properties, anti-microbial, and anti-allergic proprieties [19]. These compounds are extracted during winemaking and transmitted to the finished wine. In addition, grape pomace is one of the most abundant residues of the wine making process, with 70% of the extracted grape polyphenols remaining there. This is a valuable and cheap source of phenolic compounds and therefore of health promoting nutraceuticals [20,21]. Grape seeds may easily be separated from the pomace, and several studies have shown the possibility of obtaining grape seed oil or individual food supplements in the form of grape seed powder or grape seed extracts [22,23,24].

Although grapevine (*Vitis vinifera* L.) is considered to be adapted to semi-arid conditions, the recent predicted scenarios for global environmental change [25] suggest that despite its drought tolerance, grapevine growth in the Mediterranean area would be negatively affected in terms of berry ripening and quality. Under such a scenario irrigation emerges as a solution for grapevine cultivation. However, an appropriate balance between vegetative and reproductive development [26] is key for improving wine grape quality in irrigated vineyards. Regulated deficit irrigation (RDI) arises as one of the most promising management irrigation techniques as it has great potential to reduce vine vigour, stabilize yield and fine tune berry composition [26,27,28]. However, an understanding of berry development, and the timing for the accumulation of various components in the different berry tissues and their dependence on water availability, is critical to support an adequate irrigation program. The data already available suggest that vine water status interacts with berry development [29,30] altering metabolite accumulation, and also changing the expression of genes responsible for some grape berry compounds [26,31]. However, it has not been established whether changes in seed proanthocyanidin composition and antioxidant activity result from different irrigation practices.

The few studies that have examined the influence of water status on proanthocyanidin accumulation have reported contrasting results. Some have observed that vine water status has little impact on seed proanthocyanidin accumulation [7,32], but given that seed tannin content (mg/berry) is a linear function of both seed number and seed mass/berry [33], an impact of water availability might be expected, as reported by Cavaliere and co-authors [34]. As yet, however, no data on the impact of water deficit on the flavanol biosynthetic pathway is available.

Given the increasing interest in grape PAs due to their role in wine color and taste and their antioxidant capacity, we investigated the effect of irrigation strategy on seed flavanol monomers, and on PA accumulation, biosynthesis and anti-radical activity during berry ripening.

## 2. Results and Discussion

Although tannin content in seeds is very relevant for wine production, few studies have dealt with the effect of watering in seed polyphenols, and their results seem to be contradictory [7,33,34]. Furthermore, the accumulation of flavanols and PAs in seeds is dependent on variety [9,10,34,35]. Recent results showed that berry ripeness has an impact on the composition and extractability of seed tannins [36], and also that water deficit may affect the ripening process of berries [37,38]. In the present study we show the effect of three different irrigation systems—ranging from little stress (sustained deficit irrigation—SDI), through mild (regulated deficit irrigation—RDI) to severe (no irrigation—NI) stress—on flavanol monomer and polymeric proanthocyanidin content and biosynthesis in grape seeds during ripening.

### 2.1. Deficit Irrigation Dictates Seed Development Changes

Seed growth and number were reported to be affected [39] or to remain unchanged [7] by water stress. In the present study the seed number per berry was not significantly influenced by the irrigation regime (Table 1).

**Table 1:** Seed fresh weight (mg) and seed number in sustained deficit irrigation (SDI), regulated deficit irrigation (RDI) and non-irrigated (NI) berries during 2007 and 2008 seasons. Values are means ± SE (*n* ≥ 4). Different letters (a, b, ab) indicate significant differences among treatments at the same date using Duncan’s test (*p* ≤ 0.05).

Year	Irrigation Treatment	Seed Fresh Weight (mg)				Seed Number/Berry
		Pea Size	Veraison	Mid Ripening	Full Maturation	
2007	SDI	55.0 ± 1.0 ^a^	47.0 ± 2.0 ^a^	40.0 ± 2.0 ^a^	46.0 ± 1.0 ^b^	2.32 ± 0.1 ^a^
	RDI	52.0 ± 1.0 ^a^	55.0 ± 1.0 ^b^	50.0 ± 1.0 ^b^	42.0 ± 1.0 ^ab^	2.39 ± 0.1 ^a^
	NI	56.0 ± 1.0 ^a^	55.0 ± 2.0 ^b^	50.0 ± 1.0 ^b^	40.0 ± 0.8 ^a^	2.42 ± 0.1 ^a^
2008	SDI	56.3 ± 0.0 ^a^	60.8 ± 0.4 ^a^	60.0 ± 0.5 ^b^	47.2 ± 1.0 ^b^	1.77 ± 0.1 ^a^
	RDI	58.7 ± 0.5 ^b^	62.5 ± 0.4 ^b^	56.5 ± 0.9 ^a^	43.2 ± 0.0 ^a^	1.94 ± 0.1 ^a^
	NI	56.3 ± 0.8 ^a^	59.7 ± 0.4 ^a^	57.7 ± 0.9 ^a^	45.2 ± 0.0 ^ab^	1.92 ± 0.1 ^a^

Relative high fresh weight at pea size stage as reported in Table 1 has already been observed in several studies [7,8,40,41]. Thereafter, the fresh weight of seeds declined progressively, generally attaining the lowest values at the full maturation stage. At full maturation, seed fresh weight was lower in stressed vines (RDI and NI) compared to SDI vines (Table 1), possibly relating to a differential seed desiccation process in different irrigation treatments.

### 2.2. Water Deficit Up-Regulated Flavanol Biosynthetic Transcripts but not Flavanol Seed Content

**Figure 1:**
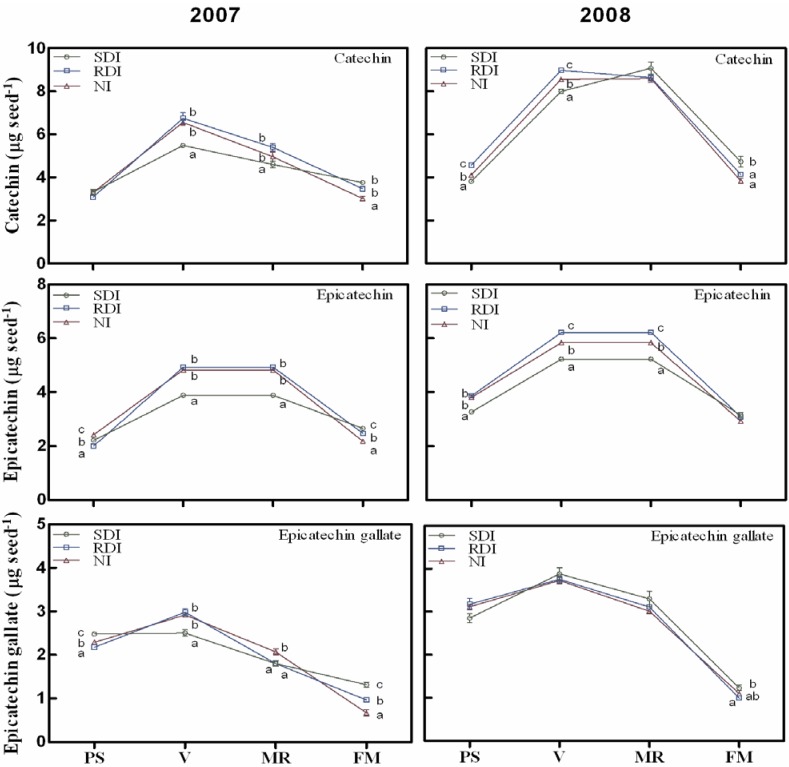
Changes in flavanol monomers during ripening of grape berry seeds in sustained deficit irrigation (SDI), regulated deficit irrigation (RDI) and non-irrigated (NI) treatments during 2007 and 2008 seasons. Values presented are means ± SE (*n* ≥ 4). Different letters (a, b, c) indicate significant differences among treatments at the same date using Duncan’s test (*p* ≤ 0.05). PS: end of pea size (7 weeks after anthesis); V: veraison (9 weeks after anthesis); MR: mid-ripening (11 weeks after anthesis); and FM: full maturation (13 weeks after anthesis).

**Figure 2:**
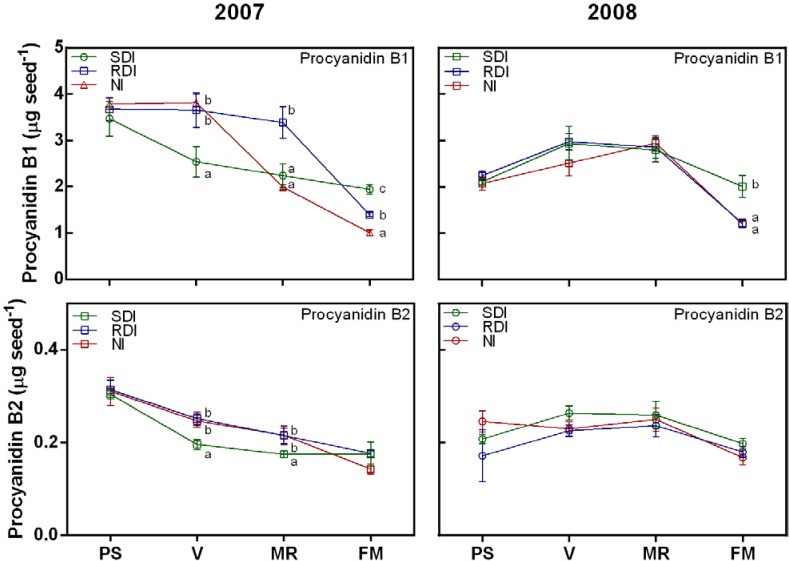
Changes in proanthocyanidins during ripening of grape berry seeds in SDI, RDI and NI treatments during 2007 and 2008 seasons.Values presented are means ± SE (*n* ≥ 4). Different letters (a, b, c) indicate significant differences among treatments at the same date using Duncan’s test (*p* ≤ 0.05). PS: end of pea size (7 weeks after anthesis); V: veraison (9 weeks after anthesis); MR: mid-ripening (11 weeks after anthesis); and FM: full maturation (13 weeks after anthesis).

Three flavanol monomers (catechin, epicatechin and epicatechin-gallate) and two proanthocyanidins (procyanidin B1 and procyanidin B2) were detected and quantified in grape seeds during both experimental years (Figure 1 and Figure 2). The general pattern of accumulation of the different compounds (monomers and procyanidins) showed an increase at the initial stages of seed development, reaching a peak at veraison and decreasing thereafter (Figure 1 and Figure 2). Two distinct periods of flavanol development in seeds have been previously described [8]; a first period in which flavanols are biosynthesized in seeds until veraison stage, and a second period in which they are modified leading to a decline in their concentration. The decrease we observed after veraison is in line with several previous reports [7,8,10,42] and may be related to a lower extraction of tannin from seeds as grapes ripen due to the conjugation of this compound with different cellular components [40] and/or due to oxidative cross-linking of polymeric tannin [8,12].

The proportion of flavanol monomers changed as seeds developed, but was maintained over the growing season regardless of the effect of water stress. Hence, the ratio catechin:epicatechin:epicatechin-gallate changed from 4:3:3 at pea size, to 5:3:2 at veraison and mid-ripening stages, to a final ratio of 5:4:1 at full maturation stage. These results contrasted in part with previous reports of Kennedy *et al*. [7,8], showing that catechin is the predominant flavonol at green stages and epicatechin the predominant compound after veraison. Their results are supported by a differential rate of decline for individual flavonol monomers after veraison. The different varieties and climate in our work may explain these differences.

The irrigation regime appears to have a deep impact on flavanol content in seeds during the two experimental years. Generally, in the first period of flavanol accumulation, RDI and NI seeds showed the highest content of both monomers and proanthocyanidin compounds (Figure 1 and Figure 2). However in both years, at full maturation, both SDI and RDI treatments increased the concentration of these compounds compared with NI berries (Figure 1 and Figure 2), corroborating a previous study with the Michele Paliere variety [34]. Some reports have shown that insufficiently ripe grapes have a higher tannin extractability [43]. Additionally, water deficit may anticipate ripening in stressed berries [37,38]. Taken together, our results suggest that seeds from irrigated berries (SDI and RDI) are less ripe than NI ones, which may explain their higher flavanol and tannins contents. In fact, seed fresh weight at the full maturation stage showed that seeds belonging to SDI vines were less desiccated than RDI and NI ones (Table 1), which may corroborate in part the unripe status of these seeds. It is also reported that water supply may modify the sugar concentration by altering sugar import and metabolism and/or water import [44], which may reduce the carbon available for carbon-based-secondary-compound biosynthesis in stressed vines and explains the reduced tannin content in RDI and NI seeds. However, different studies showed contradictory effects of water stress on flavonoid accumulation [26,30] suggesting a non-linear and complex response of berry growth and composition to water supply [44].

**Figure 3:**
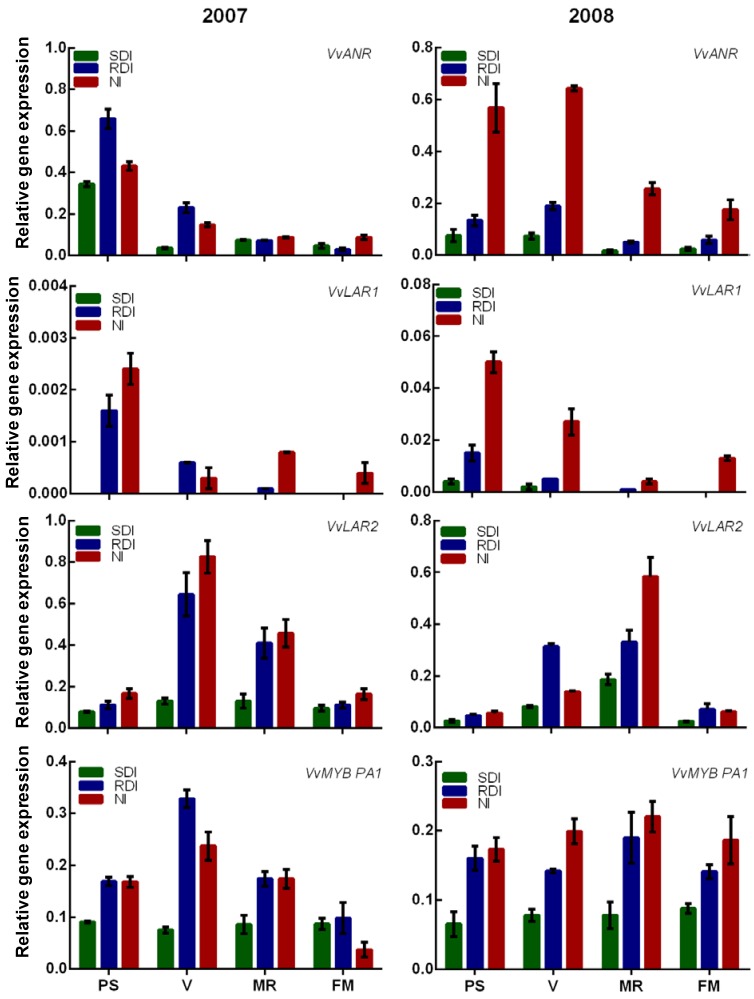
Expression of the genes *VvANR*, *VvLAR1*, *VvLAR2*, *VvMybPA1* in berry seeds of SDI, RDI and NI treatments in the seasons 2007 and 2008. Values presented are means ± SE (*n* ≥ 3). PS: end of pea size (7 weeks after anthesis); V: veraison (9 weeks after anthesis); MR: mid-ripening (11 weeks after anthesis); and FM: full maturation (13 weeks after anthesis).

As for gene expression, results suggest that flavanol biosynthesis is altered by the irrigation regime and that water stress has an impact on the extractability efficiency or/and degradation of flavanols at maturation stages. Indeed, all transcripts encoding for flavanol biosynthetic enzymes were up regulated in RDI and NI seeds at all phenological stages and both in 2007 and 2008 (Figure 3). This suggests that flavanol biosynthesis is enhanced by water stress in grape seeds, but the extraction of these compounds is probably somewhat inhibited after veraison. In contrast to Bogs *et al*. [14], who observed that the genes related to flavanol biosynthesis are no longer detected after veraison, we detected all the transcripts along berry ripening in both experimental years, except for *VvLAR1* in SDI treatment, which was undetectable in 2007 at all phenological stages and at maturation stages in 2008 (Figure 3). This occurred regardless of the decrease in the expression of some transcripts from veraison onwards. Our results could be related to varietal differences. Nonetheless, it appears that transcripts from SDI seeds showed an analogue expression, in both years, to those presented by Bogs *et al*. [14]. These findings suggest that water stress up-regulated the biosynthetic pathway of flavanol and the intensity of the stress dictate the intensity of the modification. In fact, *VvANR*, *VvLAR1* and *VvLAR2* were up-regulated in RDI and NI vine. However, NI seeds showed a higher expression of all transcripts in both years as compared with RDI ones. Bogs *et al.* [14] also showed that *VvLAR2* is the seed specific isoform, corroborating our results, since *VvLAR2* was highly correlated with catechin content both in 2007 (*r* = 0.834; *p* ≤ 0.001) and 2008 (*r* = 0.662; *p* ≤ 0.05), while no correlation was found between catechin and *VvLAR1*.

*VvMYBPA1* is a specific transcription factor controlling the expression of *VvLAR1*, *VvLAR2* and *VvANR* in grape seeds [18]. It was shown that its expression peaked at veraison and decreased thereafter. Our results showed that the expression pattern of *VvMYBPA1* was also modified in water stressed seeds (RDI and NI) compared to SDI ones, and, remarkably, the profiles established for this gene closely paralleled those of structural genes in stressed seeds (*VvLAR2* and *VvANR*). In RDI and NI vines, *VvMYBPA1* was up-regulated at all phenological stages, and its expression peaked at veraison stage in 2007 and decreased thereafter. In 2008 the high expression of *VvMYBPA1* was maintained until mid-ripening for RDI and until full-maturation for NI. It was already reported that flavanol synthesis continues in the seed up until 2–4 weeks after veraison [8], which is coincident with the expression pattern of VvLAR2 [14] and VvMYBPA1 in seeds. Both VvLAR2 and VvMYBPA1 expression reached their maximum in seeds around veraison in both years and this corresponds to the peak of flavanol monomer accumulation. Indeed, a highly significant correlation between *VvMYBPA1* and *VvLAR2* (*r* = 0.688; *p* ≤ 0.001) was found.

Altogether, these results confirm the direct effect of water stress on the flavanol biosynthetic pathway of grape seeds, which may directly affect the winemaking process and also wine quality. Research on other plant species indicates that significant changes in procyanidin biosynthesis with respect to maturity degree and cultural practices can influence the ability of procyanidins to act as astringents [45,46].

### 2.3. Antioxidant Activity Is Influenced by Irrigation Regime and Correlates with Flavanol and Proanthocyanidin (PA) Content

The antioxidant activity of seeds was assessed using two different and complementary chemical assays; oxygen radical absorbance capacity (ORAC) and hydroxyl radical adverting capacity (HORAC). These assays measure two different but equally important aspects of antioxidant properties—Radical chain breaking and radical prevention. The HORAC primarily reflects the metal chelating radical prevention ability, and the ORAC reflects the peroxyl radical absorption capacity. Results showed that both ORAC and HORAC activities increased till veraison and decreased thereafter (Table 2). The decline in both activities during the full maturation stage supports the hypothesis of the oxidation of polyphenols during seed development [8]. It is important to mention that the peak of activities differs among irrigation treatments; as an example, ORAC and HORAC were maximal at pea size stage in NI seeds, while they were maximal at veraison for RDI and SDI ones. This result support previous data on grape berry showing advanced ripening due to water stress [37,38].

**Table 2:** Oxygen radical absorbance capacity (ORAC) (µmol·TEAC·seed^−1^) and hydroxyl radical adverting capacity (HORAC) (µmol·CAE·seed^−1^) antioxidant activities in SDI, RDI and NI seeds during 2007 and 2008 seasons. Values are means ± SE (*n* ≥ 4). Different letters (a, b, ab, c) indicate significant differences among treatments at the same date using Duncan’s test (*p* ≤ 0.05).

Antioxidant Test	Year	Irrigation Treatment	Pea Size	Veraison	Mid Ripening	Full Maturation
ORAC (µmol·TEAC·seed^−1^)	2007	SDI	27.0 ± 1.2 ^a^	32.6 ± 1.7 ^b^	23.8 ± 0.8 ^b^	25.2 ± 0.1 ^c^
		RDI	28.7 ± 0.2 ^ab^	36.5 ± 1.4 ^b^	32.4 ± 2.2 ^b^	23.4 ± 0.1 ^b^
		NI	30.6 ± 0.6 ^b^	22.2 ± 1.0 ^a^	23.4 ± 1.0 ^a^	21.4 ± 0.3 ^a^
	2008	SDI	28.7 ± 2.3 ^a^	33.±2.2 ^a^	32.1 ± 1.2 ^b^	23.7 ± 1.5 ^c^
		RDI	30.5 ± 2.0 ^b^	34.5 ± 0.9 ^b^	29.3 ± 2.6 ^a^	22.9 ± 1.5 ^b^
		NI	28.6 ± 0.2 ^a^	33.2 ± 1.6 ^a^	30.0 ± 3.0 ^a^	22.1 ± 0.5 ^a^
HORAC (µmol·CAE·seed^−1^)	2007	SDI	13.4 ± 0.7 ^a^	16.0 ± 0.7 ^a^	14.4 ± 1.2 ^a^	13.7 ± 0.8 ^b^
		RDI	12.2 ± 1.0 ^a^	20.8 ± 1.3 ^b^	18.0 ± 0.8 ^a^	13.3 ± 0.6 ^ab^
		NI	19.2 ± 2.1 ^b^	15.6 ± 0.4 ^a^	15.6 ± 1.7 ^a^	10.4 ± 1.3 ^a^
	2008	SDI	16.1 ± 2.6 ^a^	18.6 ± 2.2 ^a^	17.9 ± 1.4 ^a^	15.7 ± 1.8 ^a^
		RDI	16.0 ± 2.3 ^a^	21.6 ± 1.0 ^a^	14.0 ± 3.0 ^a^	14.1 ± 1.8 ^a^
		NI	16.8 ± 0.2 ^a^	22.3 ± 2.0 ^a^	15.5 ± 3.4 ^a^	13.8 ± 0.5 ^a^

Procyanidins appeared to play a pronounced role in the ORAC antiradical activities, in particular procyanidin B1 (Table 3). Indeed, procyanidin B1 content was significantly correlated to ORAC activity in both years. In both years, ORAC correlated with all flavanol compounds being highly correlated with procyanidin B1 (Table 3). These results corroborate the work of Faria *et al*. [47] and Soobratteea *et al*. [48], which showed that the most antioxidative compound in various phenolics was procyanidin dimer. ORAC was significantly higher in SDI seeds at full maturation and in both years, followed by RDI and NI seeds. These ORAC results suggest that water availability enhanced the presence of peroxyl radicals in SDI and RDI seeds, probably by the increase of procyanidin B1 and B2 content (Figure 3). This may have several implications in winemaking, especially related to the bitterness and astringency of red wines.

**Table 3:** Correlations between ORAC and HORAC antioxidant activities and different flavanols and proanthocyanidins in SDI, RDI and NI seeds during 2007 and 2008 seasons.

Antioxidant test	Catechin	Epicatechin	Epicatechin-Gallate	Procyanidin B1	Procyanidin B2
**ORAC**	0.581 **	0.549 **	0.589 **	0.760 ****	0.467 *
**HORAC**	0.607 **	0.608 **	0.680 ***	0.443 *	0.306 ^ns^

* *p* ≤ 0.05; ** *p* ≤ 0.01; *** *p* ≤ 0.001; **** *p* ≤ 0.0001; and ^ns^ non significant.

HORAC was also significantly higher in SDI seeds in 2007. Nonetheless, in 2008 no differences were observed among irrigation treatments, indicating that additional factors may modulate the HORAC activity in grape seeds. As already reported in apple [49], HORAC correlated well with catechin, epicatechin in particular epicatechin-gallate and procyanidin B1 both in both years (Table 3).

These results indicate that in spite of only small differences between treatments in ORAC and HORAC activities, cultural practices such as irrigation can modify seed ripening and PA composition, with potential impacts for utilization of grape seeds as a source of nutraceutical compounds.

## 3. Experimental Section

### 3.1. Field Conditions and Plant Material

Grape berries were collected at four different developmental stages during the summers of 2007 and 2008 from eight-year-old grapevines of the red variety Aragonez (*Vitis vinifera* syn. Tempranillo) grafted on 1103 Paulsen rootstock from a commercial vineyard located in Estremoz, Southern Portugal. Details about the training system, plant density, ripeness (total soluble solids, titratable acidity) and leaf water potentials had been published previously [30]. The experimental layout was a randomized complete block design with three treatments and three replications per treatment. Vines were subjected to three treatments: conventional sustained deficit irrigation (SDI), regulated deficit irrigation (RDI) and Non-Irrigated (NI). The total amount of water supplied to SDI plants was 126 mm (1260 m^3^·ha^−1^) and 140 mm (1400 m^3^·ha^−1^) in 2007 and 2008 respectively, while the supply on RDI was 57 mm (570 m^3^·ha^−1^) in 2007 and 44 mm (440 m^3^·ha^−1^) in 2008. Standard cultural practices in the region were applied to all treatments. To characterize the vine water status, vine predawn leaf water potential was measured before each sampling date as described in Zarrouk *et al*. [30]. The four considered developmental stages were: (1) end of pea size (PS, 7 weeks after anthesis); (2) veraison (V, 9 weeks after anthesis); (3) mid-ripening (MR, 11 weeks after anthesis); and (4) full maturation (FM, 13 weeks after anthesis). At each sampling date a representative sample of 50 bunches per treatment was randomly collected from both sides of the vine. Samples were immediately frozen in liquid nitrogen, from which four sub-samples of 10 frozen berries each were carefully selected, peeled and the seeds removed. Seeds were weighed and ground in liquid nitrogen to fine powder and stored at −80 °C until analysis.

### 3.2. Flavanol Extraction and Analysis

Flavanol extraction from berry seeds was performed in acidified methanol. 600 µL of acidified methanol (1%) was added to 100 mg of the ground tissue, mixed 10 min at 4 °C and centrifuged at 4 °C during 15 min at 16,100× *g*. The supernatant was removed and one additional extraction was made. Both supernatants were collected and stored at −80 °C until analysis. Flavanol analysis from berry seeds was performed by HPLC–MS as described by Zarrouk *et al*. [30].

### 3.3. ORAC and HORAC Analysis

Antioxidant capacity was measured in phenolic extracts (see total phenols analysis section) by the oxygen radical absorbance capacity (ORAC) and hydroxyl radical adverting capacity (HORAC) assays.

ORAC assay was carried out using a modified method described by Serra *et al.* [50], which measures the ability of the antioxidant species present in the sample to inhibit the oxidation of disodium fluorescein, a fluorescent protein, by the peroxyl radical generator, 2',2'-azobis (2-amidinopropane) dihydrochloride (AAPH) [51]. ORAC values were calculated from the loss of fluorescence from fluorescein at different incubation time points, relative to a Trolox standard solution in similar experimental conditions and expressed as micromoles of Trolox equivalents antioxidant capacity (TEAC) per seed. All samples, including the blank and the controls, were analyzed in quadruplet.

The HORAC assay was based on a previously reported method [52], modified for the FL800 microplate fluorescence reader (Bio-Tek Instruments, Winooski, VT, USA) as described by Serra *et al*. [49]. Caffeic acid was used as a standard and data were expressed as micromoles of caffeic acid equivalents (CAE) per seed. All samples were analyzed in quadruplet.

### 3.4. RNA Extraction and qRT-PCR Analysis

Total RNA extractions were performed in a 1.5 mL tube, using the method of Reid *et al*. [53]. Total RNA was purified using an RNeasy^®^ Mini kit (Qiagen) with the addition of an on-column DNase I digestion (RNase-Free DNase Set; Qiagen, Hilden, Germany). RNA concentration was determined before and after DNase I digestion using a Nanodrop ND-1000 spectrophotometer (Nanodrop Technologies, Wilmington, DE, USA) in 260/280 nm ratio. RNA integrity was evaluated by 1% (*w*/*v*) agarose gel electrophoresis. First-strand cDNA was synthesized using the Omniscript^®^ reverse transcription kit (Qiagen, Hilden, Germany) according to the manufacturer’s instructions. The cDNA was prepared from 1000 ng of total RNA and synthesized at 37 °C for 60 min and the cDNA stored at −80 °C.

Quantitative real-time PCR was performed in the iQ5 2.0 Standard Edition (Bio-Rad, Hercules, CA, USA), sequence detection system in a 96-well reaction plate. Each reaction (20 µL) contained 250 nM of each primer, 5 µL of 1:50 diluted cDNA, and 10 µL of Power SYBR Green Master Mix (Bio-Rad). Thermal cycling conditions were 95 °C for 10 min followed by 95 °C for 10 s, 60 °C for 10 s, and 72 °C for 10 s for 40 cycles. Dissociation curves for each amplicon were then analyzed to verify the specificity of each amplification reaction; the dissociation curve was obtained by heating the amplicon from 55–95 °C. No evidence for any primer dimer or other non-specific product formation was detected for any of the primer pairs used. Each PCR was run in triplicate within the same plate, and the cycle threshold (*C*_t_) values obtained from the technical replicates were averaged. Gene transcripts were quantified by comparing the *C*_t_ of the target gene with that of actin [53]. Gene expression was expressed as mean and standard error calculated over the three biological replicates. Primer pairs for *VvLAR1*, *VvLAR2* and *VvANR* were retrieved from Bogs *et al*. [14], and *VvMYBPA1* from Bogs *et al*. [18].

### 3.5. Data Analysis

For all parameters four biological replicates were considered. Results were examined by analysis of variance (ANOVA) each season separately with SPSS software package 12.0 for Windows (SPSS Inc., Chicago, IL, USA). When the F test was significant, means were separated by Duncan’s multiple range test (*p* ≤ 0.05).

## 4. Conclusions

In this study, we found that the irrigation regime influences the flavanol biosynthetic pathway and that the different genes responsible for PA synthesis were up-regulated in the seeds of stressed grapevines (RDI and NI). The contrasted results, showing an up-regulation of flavanol biosynthesis in water stressed seeds but a decrease in their content at full maturation stage, suggest the occurrence of other mechanisms, namely, oxidation and/or degradation of PAs at late stages of maturation resulting from the impact of watering on seed ripening. The non-enzymatic antioxidant capacities (ORAC and HORAC) were modulated by water deficit and correlated well with seed PAs content, suggesting a role of water deficit not only in basic berry characteristics but also in the berry antioxidant capacity, which may ultimately be used for promoting health benefits.
